# Medical mistrust in racial minorities during the COVID-19 pandemic: Attitudes, actions and mental health outcomes

**DOI:** 10.1371/journal.pgph.0003871

**Published:** 2024-12-13

**Authors:** Pei Wang, Yutong Zhu, Zexi Jin, Wisteria Deng

**Affiliations:** 1 School of Public Health, Yale University, New Haven, Connecticut, United States of America; 2 Department of Psychology, Yale University, New Haven, Connecticut, United States of America; 3 Hackensack Meridian School of Medicine, Nutley, New Jersey, United States of America; University of Colorado Denver - Anschutz Medical Campus: University of Colorado - Anschutz Medical Campus, UNITED STATES OF AMERICA

## Abstract

Numerous studies have demonstrated that minority groups had a higher level of medical mistrust than non-minority groups, and minority communities were criticized for noncompliance with the public health guidelines during the COVID-19 pandemic. This study explores racial minorities’ attitudes and actual behavioral responses to the COVID-19 pandemic public health guidelines. A total of 221 adults responded to an online survey (mean age = 41.5; 48.0% female; 24.4% non-White). Study results indicate that racial minorities have lower trust in public health guidelines compared to non-minority groups but have taken more actions according to the public health guidelines. Analysis also uncovers the mediating roles of perceived pandemic severity and perceived public health action benefits, on the relation between minority status and public health compliance. This study contextualizes how racial minorities respond to public health crises in action, and the dissonance between that and their historical mistrust of medical authorities. This work highlights the importance of recognizing the minority’s historical burden and fostering trust in government and professionals during public health crisis.

## Introduction

### Attitudes, actions and mental health outcomes

Medical mistrust refers to the extent to which people lack confidence in the healthcare system, government health organizations, medical researchers, doctors, or other healthcare providers. It may include a person’s fear of being hurt when receiving medical treatments, concerns about the veracity or usefulness of the information provided by medical authorities, or suspicions regarding the motives of these authorities [1, 2]. Prior scientific work has reported minority groups (such as Black Americans) as having a high mistrust of medical authorities [3, 4], and such hesitation is not unfounded. For example, historically, doctors and other medical professionals have subjected Black Americans to various forms of medical mistreatment, including subjecting them to medical experiments without their informed consent [5, 6]. Doctors gave untested medications and unapproved vaccines to enslaved Black Americans as trials [6]. During the antebellum era (1815–1861), medical institutions acquired black bodies by kidnapping or denying medical service to diseased black patients for autopsy education [7].

Racially segregated health care was established at the beginning of the slavery era [8]. During this period (17^th^-19^th^ century), a large number of scientists claimed that black people were naturally suited for slavery and that their emancipation would result in disease and death [8]. In the 1830s, doctors subjected enslaved Black people to the majority of experimental surgeries [5–7]. Historical injustice led to the Tuskegee Syphilis Study, a key event fueling Black Americans’ mistrust of U.S. medical care. In this study, the researchers did not inform or treat the Black American participants with syphilis of their sickness [3, 5, 7]. Medical maltreatment of Black Americans has continued to plague modern medicine. For instance, Black patients receive pain medication less frequently because physicians today still have the perception that Black patients can tolerate more pain [3].

Given the long history of medical mistreatment, it is easy to understand Black Americans’ ingrained mistrust of medical authorities. Medical malpractice and racial discrimination have not ceased to date and continue to erode their trust in medical authorities. According to the 2016 study by the National Urban League, the level of equality for Black Americans has grown in several areas, including health, education, economy, civic involvement, and social justice. Nonetheless, Black Americans’ mistrust of medical authorities persists, revealing the sustained impact of historical mistreatment and unresolved psychological burden [7].

The long history of medical mistrust extends beyond the Black community and have lasting impacts in a multitude of racial and ethnic minority groups. One survey has revealed that Hispanic adults have 49% higher chances of reporting medical mistrust than non-Hispanic White adults [9]. Another study has also shown that Hispanic patients lack trust in medical professionals, and they are twice as likely as White patients to fear being exploited as test subjects [10]. Additionally, Native American cancer patients have demonstrated less trust and satisfaction with healthcare providers and hospitals than non-Hispanic White patients [11]. Prior research also revealed a general mistrust in Western healthcare system and medical practices among the Asian Americans [12, 13]. All in all, fueled by historical fear and cultural differences, medical mistrust is a common theme across racial minority groups.

### Medical mistrust during the COVID-19 pandemic

Wuhan, China experienced an outbreak of pneumonia of unclear origin in December 2019. On March 12, 2020, the World Health Organization declared a public health emergency of international concern (PHEIC) due to the global spread of SARS-CoV-2 and thousands of deaths due to coronavirus disease (COVID-19). To date, the pandemic has cost countless human lives, negative economic impacts, and rising levels of poverty [14]. There have been over 98.5 million confirmed cases and over 1.1 million deaths in the United States [15]. During this pandemic, the U.S. public has faced numerous challenges, such as incompetent responses from government officials and unclear COVID-19-related health messaging. The government’s inconsistent narrative about COVID-19 and its preventative measures has generated a great deal of uncertainty and anxiety [1, 16]. The plague of misinformation on social media has also negatively impacted vaccine hesitancy and uptake. Unfound rumors about COVID-19’s development and vaccine impacts have spread so quickly on social media that the World Health Organization coined the term “infodemic,” highlighting the amount of incorrect or misleading information online and in real life during this pandemic [17]. “Infodemic” further eroded the already frail medical trust among racial minorities. For instance, racial minority groups, particularly the Asian American communities, faced heightened racism and xenophobia during the COVID-19 pandemic, fueled by disinformation endorsed by political elites that framed Asian Americans as disease spreaders [18, 19].

Early in the COVID-19 pandemic, in February 2020, news outlets, politicians from different political parties, and medical professionals stated that COVID-19 was not likely to be a serious threat in the U.S. The narrative was challenged in late March 2020, as the U.S. reported the most confirmed COVID-19 cases around the world [20]. Conspiracy theories about COVID-19 as manufactured by the federal government circulated on social media, demonstrating a prevalent mistrust in the government and medical authorities [3, 21]. A nationally representative survey done on April 2020 found that almost one-tenth of adults did not expect to receive vaccine against COVID-19, and three-tenths of adults were uncertain about accepting vaccination [22]. Besides discouraging or preventing individuals from accepting COVID-19 vaccination, medical mistrust may also dissuade people from accessing medical treatment related to COVID-19 or following evidence-based preventative measures, such as social distancing and self-quarantining [1].

The World Health Organization has reaffirmed that COVID-19 vaccinations are essential and effective against death and serious illness caused by the coronavirus. However, after the COVID-19 vaccine Pfizer-BioNTech phase III clinical trial in November 2020, conspiracy claims, medical misinformation, and concerns about the quick vaccine development have lingered and fed into the public’s vaccine reluctance [17]. Conspiracy claims stated that the COVID-19 vaccine contains microchips that allow governments to control people. For others, the fear of side effects significantly contributed to the reluctance of individuals to be vaccinated. Social media has frequently misrepresented the side effects of vaccinations, including infertility, chronic disease, alterations in DNA, physical abnormalities, and mental illness [17, 23]. As reported by CDC in 2024, the vaccine uptake rate was still low [24, 25].

### Racial minorities during the COVID-19 pandemic

The pandemic has disproportionately impacted racial minorities. The CDC has reported that by the end of 2022, non-Hispanic White people had lower rates of COVID-19 cases, hospitalization, and deaths compared to Black Americans, Hispanic Americans and Native Americans [26]. Research has attributed the higher mortality among these minority groups to a significantly higher vaccine reluctance rate [1]. Indeed, a study has revealed that the overall COVID-19 vaccine reluctance for adult Americans is 26.3%, while the hesitancy for Black Americans is 41.6% and that for Hispanic Americans is 30.2% [27]. Prior work also showed that Black people have the highest vaccine trial participation rejection rate compared to other ethnic groups [1]. Only 18% of Black Americans and 40% of Hispanic Americans trust the efficacy of the COVID-19 vaccine. Even fewer–only 14% of Black Americans and 34% of Hispanic Americans–trust the safety of the vaccine. 28% Black Americans and 47% Hispanic Americans believe that the vaccine’s safety will be examined for their racial group [28, 29]. Even if the vaccine is free and scientifically proven to be safe, less than 20% of Black Americans reported a “definite” willingness to have vaccination. In contrast, 37% White people claim they would “definitely” get vaccinated [30].

Besides concerns about the safety of the vaccine, medical mistrust has also interfered with vaccination willingness among Black, Hispanic, and Asian Americans. Individuals with higher medical mistrust are less likely to receive COVID-19 vaccinations [31]. In comparison to White adults, Black Americans’ trust in doctors is 19 percent lower, trust in local hospitals is 14 percent lower, and trust in the health care system is 11 percent lower. Similarly, Hispanic Americans’ trust in doctors is 6 percent lower, trust in local hospitals is 8 percent lower, and trust in the health care system is 5 percent lower [30]. Asian Americans reported similar medical mistrust as the Hispanic and the Black communities [31].

### The Health Belief Model

The Health Belief Model (HBM), initially developed in the 1950s, is a framework that helps predict individual behavior in relation to disease detection, prevention, and control. This model consists of six key constructs: perceived susceptibility, perceived severity, perceived benefits, perceived barriers, self-efficacy, and cues to action [32–34]. Perceived severity encompasses people’s understanding of the consequences of contracting a particular condition. Perceived benefits involve individuals’ perception of the potential advantages associated with taking recommended actions. According to the HBM, individuals are more likely to take actions if they perceive themselves as susceptible to a condition, recognize its severity, believe that taking advised actions will benefit them, perceive manageable barriers, and have confidence in their ability to act [33, 34]. However, the exact relationship and order of these constructs are not clearly defined, especially in the case of COVID-19 pandemic [32, 35].

### The present study

Much evidence indicates that the COVID-19 pandemic has disproportionately harmed Black Americans and other minority groups, and minority status is a predictor of people’s reactions to the pandemic [4, 22]. Whereas existing studies show that racial minorities have more negative attitudes toward medical authorities, and that they report more reluctance to accept vaccinations against COVID-19, researchers have done little work to extend our knowledge beyond their reported attitudes and capture the actual behaviors. Indeed, reported attitudes may not necessarily reflect individuals’ behaviors, including the degree to which they adhere to public health guidance. The assumption that negative attitudes toward medical authorities are equivalent to risky public health decisions is unfair and may mistakenly put the minorities at fault.

The present study investigated minorities’ reported attitudes and actions regarding public health guidelines during the pandemic to determine if there is a discrepancy between minorities’ attitudes and public health behavior. The public health experiences of racial minorities are shaped by more than race alone. The intersectionality theory suggests that individuals who belong to both racial and sexual minority groups, may encounter unique stressors and health outcomes that exceed the simple addition of stressors associated with each minority identity [36]. By including both racial and sexual orientation data, we sought to capture the intersecting forms of stress racial minority individuals may experience, which can impact their health-related attitudes and behaviors. In this study, we hypothesized that racial minority individuals would report less trust in government officials and health professionals during the pandemic, while still adhering to the public health advisory as much as the rest of the population. We aimed to uncover the potential misalignment between minority individuals’ self-reported attitude and their behaviors in response to public health guidance during the pandemic and further explore the factors that impact minority’s public health behaviors. Differentiating between attitudes and behaviors can help us better understand how minorities respond to a public health crisis. Our work also aims to reiterate the past unjust medical treatments and sustained mistrust within the racial minority individuals, in order to boost the effectiveness of our public health guidance and intervention during the pandemic.

## Materials and methods

### Procedure and participants

Amazon’s Mechanical Turk (Mturk), an online crowdsourcing platform that gives researchers access to a sizable and varied sample for mental health research studies, was used to recruit participants (*N* = 221, *M*_*age*_ = 41.18, *SD*_*age*_ = 12.09; Demographics see S1 Table). Users of Mturk who reside in the United States and are 18 years of age or older could participate in the study. In accordance with recommendations for research using crowdsourced samples, the study only enlisted Mturk users who have a history of giving high-quality responses [37]. Each participant is required to finish at least 500 Mturk studies, with 98% of those studies accepting their answers. Participants completed a questionnaire that began with questions about their demographic information and continued with ratings of their attitudes and behaviors in accordance with the COVID-19 public health guidelines. Then, the participants then completed the discrimination-related questions. After completing the survey, participants received a debriefing and compensation of $6.

### Measures

#### Minority status

Racial and sexual orientational statuses were collected from the participants. Participants reported to be heterosexual *and* White were coded 1 (“no”) in this variable; participants reported to be non-heterosexual *or* non-White were coded 2 (“yes”).

#### Attitudes and actions during the COVID-19 pandemic

Self-report measures regarding the pandemic were also collected to examine individuals’ attitudes toward the authorities and behavioral responses during the COVID-19 pandemic. Participants’ trust in the leadership and public health professionals were rated using two questions. Participants’ level of trust was rated on a four-point Likert scale, from 0 to 3. Their perception of the severity of the pandemic was also rated under a four-point Likert scale, from 0 to 3. In addition, participants were asked to report to what extent they think taking specific actions to prevent the spread of COVID-19 is important, and the number of actions taken to protect themselves. Question details are presented in S1 File.

#### Discrimination

The 9-item Everyday Discrimination Scale (EDS) measures the frequency of daily microaggression or discrimination [38]. Each item includes a six-point Likert scale with the values 0 (“never”) to 5 (“nearly every day”). The EDS also inquiries about the respondent’s perceptions of the primary “causes” of their discriminatory experiences. There is strong construct validity for the EDS among ethnic minority groups, according to research [39].

### Statistical analysis

The statistical analyses were conducted using RStudio Mac Version 2023.03.0 (Copyright © 2022 by Posit Software, PBC). Descriptive analyses were first performed to explore potential correlations between the research variables. Independent sample t-tests and ANOVAs were then used to examine mean differences between discrimination and COVID-19-related behavioral measures based on the participant’s minority status. The significance level was set at 0.05 for two-tailed p-values.

To investigate potential mediating effects among the study variables, mediation analyses were performed using Macro PROCESS Version 4.3. Model 4 and Model 6 analytical procedures were employed to explore the relationships proposed in the hypothesized models. The independent variable in the model was minority status, while the dependent variable was the number of health-related actions taken. To examine the mediation pathways, serial mediation effect of perceived pandemic severity and perceived health measure benefits was examined in that order (Model 1) and reversed (Model 1 alternative). The mediating impact of perceived barriers include trust in government (Model 2), trust in experts (Model 3), and discrimination (Model 4) were also assessed. See S1 Fig for figures of Model 2, Model 3, and Model 4. The significance of the mediation effects was evaluated by conducting 5,000 bootstrap samples and examining the 95% confidence intervals. Moreover, covariates including age, gender, education, marital status, and household income were incorporated in all models to account for their potential influence on the investigated relationships.

### Ethics statement

Ethical approval was obtained from Yale University’s Institutional Review Board. In compliance with Yale University’s Institutional Review Board, formal written consent were obtained from all participants. The recruitment started on January 20^th^, 2023 and ended on June 5^th^, 2023.

## Results

### Descriptive statistics

Greater trust in government was highly associated with greater trust in experts (*r* = .676, *p* < .001), less perceived pandemic severity (*r* = -.328, *p* < .001), less perceived health measure benefits (*r* = -.437, *p* < .001), and fewer actions taken during the pandemic (*r* = -.287, *p* < .001) (Table 1). Perceived pandemic severity, benefits, and reported actions taken were highly correlated with each other: higher perceived severity was associated with higher perceived health measure benefits (*r* = .520, *p* < .001) and more actions taken in response to the pandemic (*r* = .509, *p* < .001). Discrimination was highly associated with greater perceived severity (*r* = .223, *p* < .001) and more health-related actions taken (*r* = .136, *p* = .043), but not perceived benefits (*r* = -.012, *p* = .861).

**Table 1:** Descriptive statistics and correlations of study variables.

Variables	*M*	*SD*	1	2	3	4	5	6
1. Trust in Government	1.40	.897	-					
2. Trust in Experts	.92	.868	.676***	-				
3. Perceived Pandemic Severity	1.35	.655	-.328***	-.336***	-			
4. Perceived Benefits	2.76	1.162	-.437***	-.501***	.520***	-		
5. Reported Actions Taken	7.88	3.096	-.287***	-.330***	.509***	.439***	-	
6. Discrimination	20.51	11.112	.017	.062	.223***	-.012	.136*	-

*N* = 221

*p < 0.05

**p < 0.01

***p < 0.001.

### Minority status and COVID-19-related behavioral measures

Independent sample t-tests suggest that the minority group did not report significant differences in trust in the government (*t* = 1.620, *p* = .107) or expertise (*t* = 1.059, *p* = .291) compared to the non-minority group. The minority group reported higher perceived severity of the pandemic (*t* = -3.060, *p* = .003) and higher perceived benefits of public health guidelines (*t* = -2.138, *p* = .034). However, the minority group also reported taking more actions in line with the public health guidance to protect themselves (*t* = -2.393, *p* = .018) during the pandemic (Table 2).

**Table 2:** Minority status differences in COVID-19-related behavioral measures.

Variables	Minority Group *M (SD)* (*n =* 83)	Non-minority Group *M (SD)* (*n =* 138)	*t*	*p*	*Cohen’s d*
Trust in Government	1.28 (.846)	1.48 (.922)	1.620	.107	0.225
Trust in Experts	.84 (.848)	.97 (.879)	1.059	.291	0.147
Perceived Pandemic Severity	1.53 (.704)	1.25 (.602)	-3.060	.003**	0.442
Perceived Benefits	2.96 (1.076)	2.63 (1.197)	-2.138	.034*	0.289
Reported Actions Taken	8.52 (2.993)	7.50 (3.105)	-2.393	.018*	0.332

*N* = 221

*p < 0.05

**p < 0.01

***p < 0.001.

### Role of individual’s attitude and trust in the association between minority status and health-related actions taken

**Fig 1:**
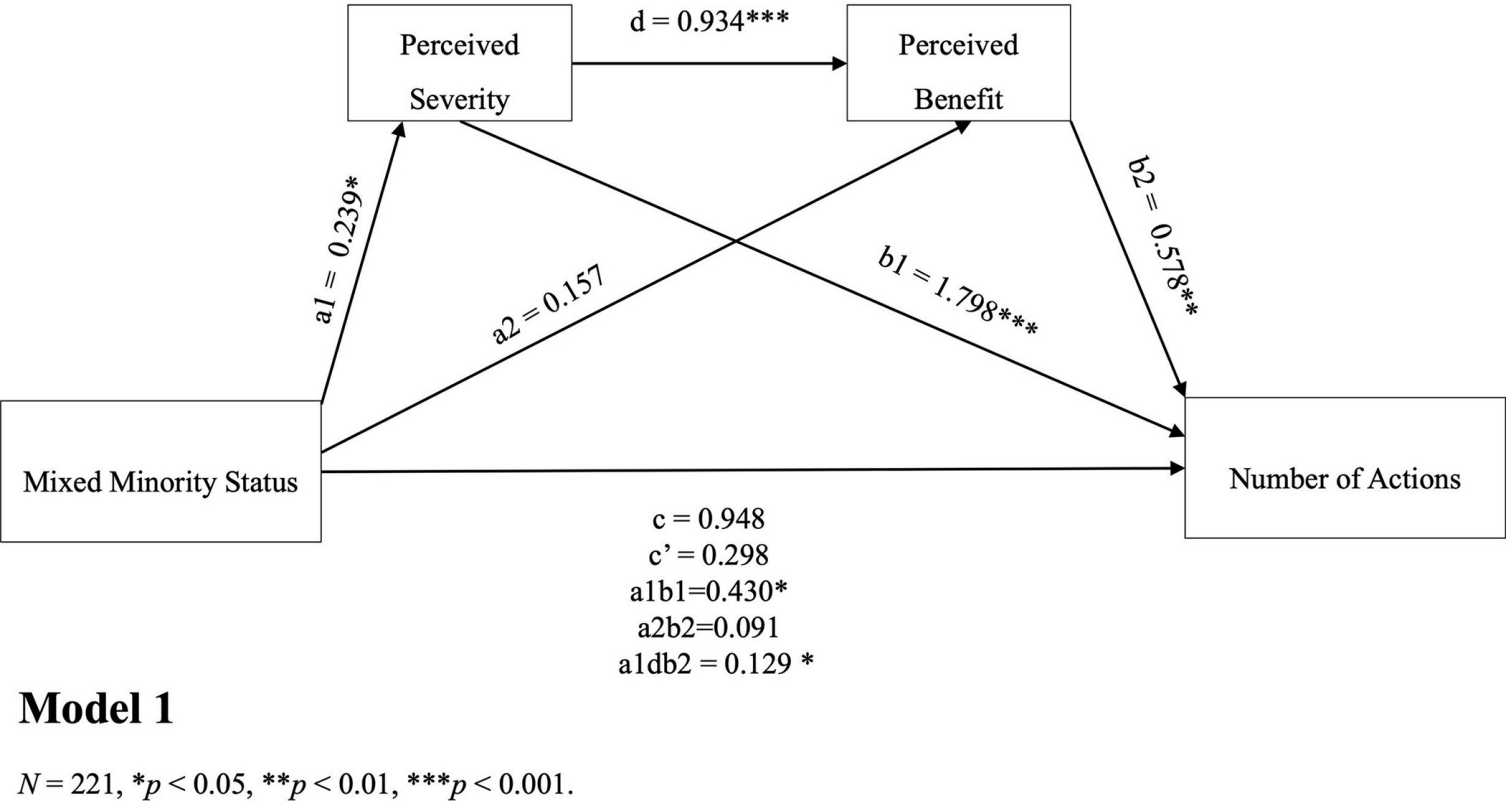
Perceived severity and perceived benefit as serial mediators.

**Fig 2:**
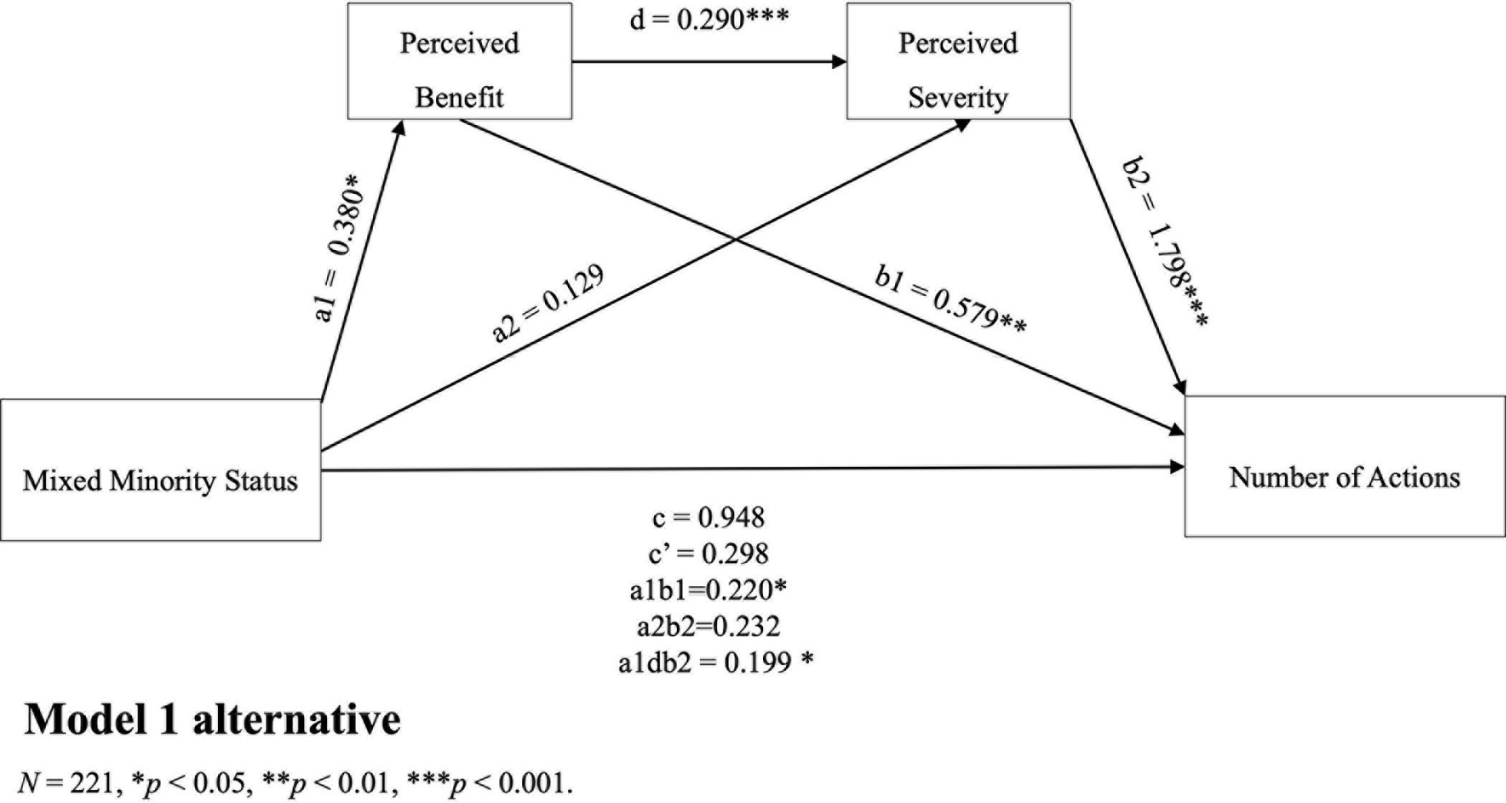
Perceived benefit and perceived severity as serial mediators.

The results of the mediation models indicated that there were indirect effects of minority status and health-related actions taken through perceived severity and perceived benefit as serial mediators (*β* = 0.129, 95%CI [0.074,0.306]). The findings revealed that perceived severity and perceived benefit combined fully mediated the relationship between minority status and the number of health-related actions taken by individuals. When perceived severity was considered the first mediator, the analysis confirmed that perceived stress independently mediated the relationship (a1b1 = 0.430, BootCI [0.083, 0.829], Fig 1). Moreover, the association between perceived stress and the reported number of actions remained significant even after controlling for perceived benefits (b1 = 1.798, 95%CI [1.169, 2.426]). Importantly, there was a significant association between the mediators, with minority status and perceived severity explaining 30% of the variance in perceived benefits (*F*(7, 213) = 13.13, *p* < 0.001, *R2* = 0.301), after accounting for all covariates. To explore the potential directionality between perceived severity and benefit, we conducted a new analysis by reversing the order of the mediators (Fig 2). The results from the reversed model supported our previous finding that perceived benefits acted as an independent mediator (a1b1 = 0.220, BootCI [0.023, 0.505]). Also, higher perceived benefits were associated with higher perceived severity, which, in turn, was associated with a greater number of health-related actions. Controlling for all covariates, the mediators remained significantly associated, with minority status and perceived benefits explaining 32% of the variance in perceived severity (*F*(7, 213) = 14.16, *p* < 0.001, *R2* = 0.318).

However, discrimination did not show a significant mediating effect on the association between minority status and reported number of actions taken (*β* = 0.116, 95%CI [-0.034,0.336]). No significant mediating effect were found for trust in experts (*β* = 0.148, 95%CI [-0.106,0.429]). Trust in government did not have significant association with minority status (*β* = -0.252, *p* = 0.054), so it did not have a significant mediating effect for minority status on number of health-related actions. Detailed coefficients can be found in S2 Table.

### Trust in government but not in experts moderates the relationship between perceived severity and benefit

**Fig 3:**
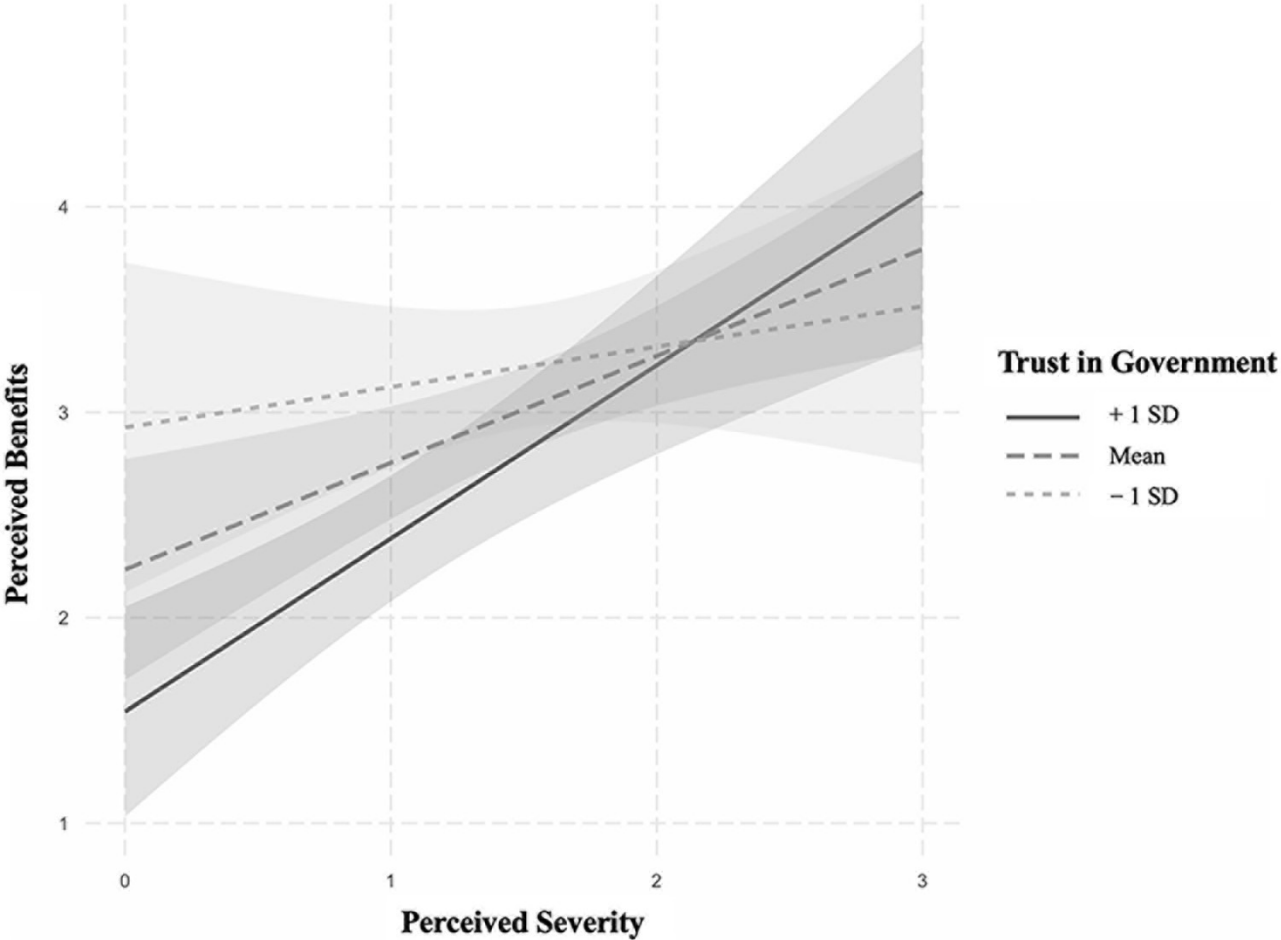
Interaction effects of perceived pandemic severity and trust in government on mixed minority individuals’ perceived benefits (n = 83). Age, gender, education, marital status, and household income were included as covariates.

We further explored trust in government and trust in experts as moderators between perceived severity and benefit. The result revealed a significant main effect of trust in government on perceived benefits (*β* = -0.818, 95%CI [-1.297, -0.340], *p* = 0.001), while insignificant main effect of perceived severity on perceived benefits (*β* = 0.031, 95%CI [-0.578,0.641], *p* = 0.919) among the mixed minority group (*n* = 83). The moderation analysis results demonstrated that trust in government had a significant moderating effect on the association between perceived severity and benefit (*β* = 0.382, 95%CI [0.038,0.727], *p* = 0.030, ΔR^2^ = 0.042, Fig 3). Further examination of the conditional effects of perceived severity was conducted at three levels of trust in government: mean-1SD, mean, and mean+1SD. The findings indicated a significant relationship between perceived severity and perceived benefits at all three trust levels (p values ranging from 0.022 to < .001). Additionally, a 5,000-sample bootstrap test confirmed the significant moderating effect of trust in government. Therefore, individuals from mixed minority backgrounds who exhibited higher levels of trust in government experienced a stronger influence of perceived pandemic severity on perceived benefits.

In contrast, the result indicated a significant main effect of trust in experts and perceived severity on perceived benefits (*β* = -0.506, 95%CI [-0.948,-0.065], *p* = 0.025 and *β* = 0.516, 95% CI [0.090,0.942], *p* = 0.018 respectively) when trust in experts was included as the moderator instead of trust in government. However, the moderation analysis did not demonstrate a significant moderating effect of trust in experts on the association between perceived severity and benefit (*β* = 0.075, 95%CI [-0.231,0.382], *p* = 0.625, ΔR^2^ = 0.002). Thus, the level of trust that mixed minority participants placed in experts did not influence the relationship between perceived severity of the pandemic and perceived benefits.

## Discussion

This paper revealed the gap between reported attitudes and actual behavioral responses in following the public health guidelines during a pandemic. Specifically, this study discovered that while racial minorities reported less trust in the public health guidelines during the COVID-19 pandemic, they still adhere to such advisory as much as others, even taking more preventative actions in response to the pandemic. The mediation analysis suggested that the association between minority status and behavioral responses in following public health guidelines during a pandemic was mediated by perceived pandemic severity and perceived health measure benefits. Furthermore, trust in government showed a moderating role in the relationship between perceived pandemic severity and perceived health measure benefits. This implies that the perceived severity of the pandemic and the perceived benefits of public health measures (e.g., vaccine, social distancing) can help explain why minority individuals exhibit a high level of reported adherence to public health guidelines, despite having a lower level of trust in the public health authorities. Additionally, this study also exhibited differing effect of perceived barriers. In this case, only trust in government had the modifying impact on the association between perceived severity and perceived benefits among the minority individuals. The existence of multiple possible paths through which the effect of minority status is transmitted to public health compliance (i.e., reported number of actions taken in response to public health guidance) is in line with the complexity of multiple factors influencing health beliefs, as suggested by the HBM [32–34]. The divergence between racial minority individuals’ reported attitude and their behavioral responses indicates a dissonance between their beliefs and actions. Prior work on minority stress has shown such dissonance in other minority groups. For example, sexual and gender minorities who also belong to religious communities reported cognitive dissonance between their religious practices (which may involve anti-LGBTQ messaging) and their beliefs (centered around protecting their identity) [3, 4]. Racial minorities who experience a conflict between their traditional values and the societal norm may also report psychological discomfort [40]. Based on the cognitive dissonance theory, an underlying tension is created when an individual’s behavior is inconsistent with their thoughts. To resolve such tension, an individual has to form new beliefs about the self in order to justify their actions [41]. Having to follow the public health guidelines when there is still unresolved mistrust may prompt negative beliefs about the self in order to justify the actions and decrease cognitive dissonance. As explanations for “following the orders”, an individual may form negative view of the self (e.g., lower self-esteem), negative view of the world and the future (e.g., hopelessness). Coupled with the heightened psychological distress during the pandemic, these negative beliefs can lead to adverse mental health outcomes for minority individuals.

Our work accentuates the importance to reflect on public narratives during a pandemic. Prior studies have warned of the danger of scapegoating minorities during a public health crisis [42–44]. For example, numerous research has revealed a rise in anti-Asian sentiments during the COVID-19 pandemic and the subsequent negative health outcomes [45]. Arabic-named women also had worse birth outcomes six-month following the occurrence of 911 in 2001 compared to before the event due to increased race-specific discrimination and blaming [46]. Many studies have focused on minorities lacking trust in public health authorities and not adhering to public health guidelines, and there have also been discussions about addressing minority groups’ trust in the medical authorities through education and highlighting the importance of improving people’s attitude toward vaccination against COVID-19 through more consistent and accurate public health guidelines [7, 16]. However, researchers based most of these finding on reported attitudes instead of behavioral responses. It is worth considering cognitive dissonance, and differentiating attitudes from actions among minority individuals, as they cope with frequent discriminations and other chronic stressors. Future research can also strive to obtain a more comprehensive understanding of racial minorities’ response to public health crises through various aspects. Beyond the scope of our study, understanding the influence of disinformation on racial minorities during public health crises is also crucial for future studies. Disinformation can negatively impact individual’s health, and contribute to societal instability [47, 48]. Future research should explore the key sources of disinformation that contribute to the stigmatization of marginalized populations during public health crises. It is also important to identify key stakeholders, such as policymakers, public health professionals, and community leaders, to enhance the effectiveness and accuracy of messaging to combat the spread and negative impact of misinformation and disinformation and better prepare for future public health emergencies.

This study is not without limitations. Different racial minority groups experience unique sociocultural factors that impact their health-related stress. For instance, Black Americans have a long history of being subjected to unethical medical experimentation, which continues to affect their trust in healthcare systems [7]. Asian Americans, influenced by Confucian values that discourage public admission of illness, coupled with traditional beliefs in herbal healing, may approach healthcare differently. Lower income levels among Hispanics, compared to both Black and White Americans, limit their access to healthcare services and health-related practices. Lastly, the diversity within Native American communities complicates the understanding of their health practices, making them even more understudied than other racial groups [49]. While we still have a sufficient sample size of minority participants to make a statistically significant comparison against heterosexual and White participants, the study may not be able to generalize to specific racial minority groups as the imbalance in sample sizes across racial groups could possibly skew the results. This limitation is mainly due to the difficulty of selective sampling through online crowdsourcing platforms because racial minorities have a lower participation rate in web-based surveys compared to non-minority groups [50]. Similarly, people with lower socioeconomic status may be less likely to participate in this study, as they have less access to internet services [51], resulting in a less representative sample of the broader population. Future studies may consider focusing on the inclusion or oversampling of more diverse participants using a community-based recruitment approach to better understand heterogeneity among individuals with one or multiple minoritized statuses. In addition, while the study covered important facets of subjective attitudes towards public health responses, the survey only captured concerns during the early phase of the pandemic. Future studies might be able to retrospectively measure how individuals’ attitudes evolved throughout different phases of the pandemic, especially in relation to the roll-out of various public health measures. Third, while our studies focused on attitudes and behavioral responses among racial minorities, other marginalized groups such as those with lower education levels and lower socioeconomic status are also subject to medical mistrust [52]. Future research can investigate how overlapping minority identities within racial minority groups impact the dissonance between attitudes and behavioral responses toward public health guidelines. Although self-report measures are commonly used in research and allow for efficient data gathering, the study’s use of self-report questionnaires may introduce the possibility of self-reporting bias as respondents may alter their responses for reasons such as social desirability, leading to underreporting or overreporting to the measures of the study’s interest [53]. Future studies could use a combination of self-report measures and objective measures, like medical records, to mitigate the impact of self-reporting bias.

Despite the limitations, our study holds significant implications for public health, emphasizing the importance of not scapegoating minority groups for the severity of public health crises. It is crucial to acknowledge that various factors and complex pathways contribute to public health attitudes and behaviors. To improve mental health outcomes among the racial minorities, it is not only important to strive toward quantifiable goals (e.g., wealth and resource allocation), but also integral to address the underlying psychological burden by reflecting on the historical context. In a healthcare setting, this means not to discuss any individual experience in a vacuum, but to educate medical providers in recognizing the historical burden and other environmental stressors experienced by the minority individuals. It is also imperative for the government to foster trust and credibility, assuming a role as a trustworthy source, and effectively communicate accurate information regarding the severity of pandemics and benefits of public health guidelines.

## Supporting information

S1 Table Demographic characteristics of participants. (DOCX)

S2 Table Mediation analysis output for Model 1–4. (XLSX)

S1 Fig Proposed Model 2–4 with trust in government, trust in experts, and discrimination as mediators between mixed minority status and number of actions taken. (PDF)

S1 File Attitudes and actions during the COVID-19 pandemic questionnaire. (PDF)
