# Supplementary material for: Medical mistrust in racial minorities during the COVID-19 pandemic: Attitudes, actions and mental health outcomes
Source: PLOS Glob Public Health. 2024 Dec 13;4(12):e0003871. doi: 10.1371/journal.pgph.0003871 (PMC11642957; doi:10.1371/journal.pgph.0003871)
Supplement: S1 Table — (DOCX) [file pgph.0003871.s001.docx]

**Demographic Characteristics of Participants.**

| Gender | |  | | # | % |  | Psychiatric History | | # | % |
| --- | --- | --- | --- | --- | --- | --- | --- | --- | --- | --- |
|  | Male  (Mage = 41.25, SD = 12.56) | | | 115 | 52.04% |  |  | Yes | 55 | 24.89% |
|  | Female  (Mage = 41.09, SD = 11.61) | | | 106 | 47.96% |  |  | No | 166 | 75.11% |
| Sexual Orientation | | | |  |  |  | Psychiatric Medication History | | |  |
|  | Heterosexual | | | 182 | 82.35% |  |  | Yes | 47 | 21.27% |
|  | Homosexual | | | 5 | 2.26% |  |  | No | 174 | 78.73% |
|  | Bisexual/Pansexual | | | 26 | 11.76% |  | Current Medication (if applicable) | | |  |
|  | Asexual/Aromantic | | | 7 | 3.17% |  |  | Mental health | 33 | 56.90% |
|  | Queer/Questioning | | | 1 | 0.45% |  |  | Medical problems | 27 | 46.55% |
| Race/Ethnicity | | | |  |  |  |  | General health | 3 | 5.17% |
|  | White/Caucasian | | | 167 | 75.57% |  | Family History of Mental Illness | | |  |
|  | Black/African American | | | 23 | 10.41% |  |  | Yes | 61 | 27.60% |
|  | Asian | | | 15 | 6.79% |  |  | No | 160 | 72.40% |
|  | Native American/Indigenous | | | 1 | 0.45% |  | Employment | |  |  |
|  | Others | | | 15 | 6.79% |  |  | Currently employed, full-time | 159 | 71.95% |
|  | Hispanic/Latino | | | 32 | 14.48% |  |  | Currently employed, part-time | 28 | 12.67% |
| Marital Status | | | |  |  |  |  | Student | 3 | 1.36% |
|  | Single/Never married | | | 78 | 35.29% |  |  | Retired | 7 | 3.17% |
|  | Currently married | | | 88 | 39.82% |  |  | Unable to work | 3 | 1.36% |
|  | Divorced | | | 21 | 9.50% |  |  | Stay at home parent | 5 | 2.26% |
|  | Separated | | | 2 | 0.90% |  |  | Currently unemployed, looking for employment | 11 | 4.98% |
|  | Currently in a relationship but not married | | | 32 | 14.48% |  |  | Currently unemployed, not looking for unemployment | 5 | 2.26% |
| Educational Level | | | |  |  |  | Household Income | |  |  |
|  | Less than high school | | | 1 | 0.45% |  |  | Less than $20,000 | 21 | 9.50% |
|  | High school | | | 15 | 6.79% |  |  | $20,000 to $34,999 | 20 | 9.05% |
|  | Some college | | | 40 | 18.10% |  |  | $35,000 to $49,999 | 45 | 20.36% |
|  | 2-year degree/Associate's degree | | | 28 | 12.67% |  |  | $50,000 to $74,999 | 53 | 23.98% |
|  | 4-year degree/Bachelor's degree | | | 105 | 47.51% |  |  | $75,000 to $99,999 | 38 | 17.19% |
|  | Master's degree | | | 27 | 12.22% |  |  | $100,000 to $149,999 | 28 | 12.67% |
|  | Doctorate | | | 5 | 2.26% |  |  | $150,000 to $199,999 | 9 | 4.07% |
| Native Language | | |  |  |  |  |  | $200,000 or more | 7 | 3.17% |
|  | English | | | 218 | 98.64% |  |  |  |  |  |
|  | Others | | | 3 | 1.36% |  |  |  |  |  |
