# Supplementary figures and images for: Medical mistrust in racial minorities during the COVID-19 pandemic: Attitudes, actions and mental health outcomes
Source: PLOS Glob Public Health. 2024 Dec 13;4(12):e0003871. doi: 10.1371/journal.pgph.0003871 (PMC11642957; doi:10.1371/journal.pgph.0003871)

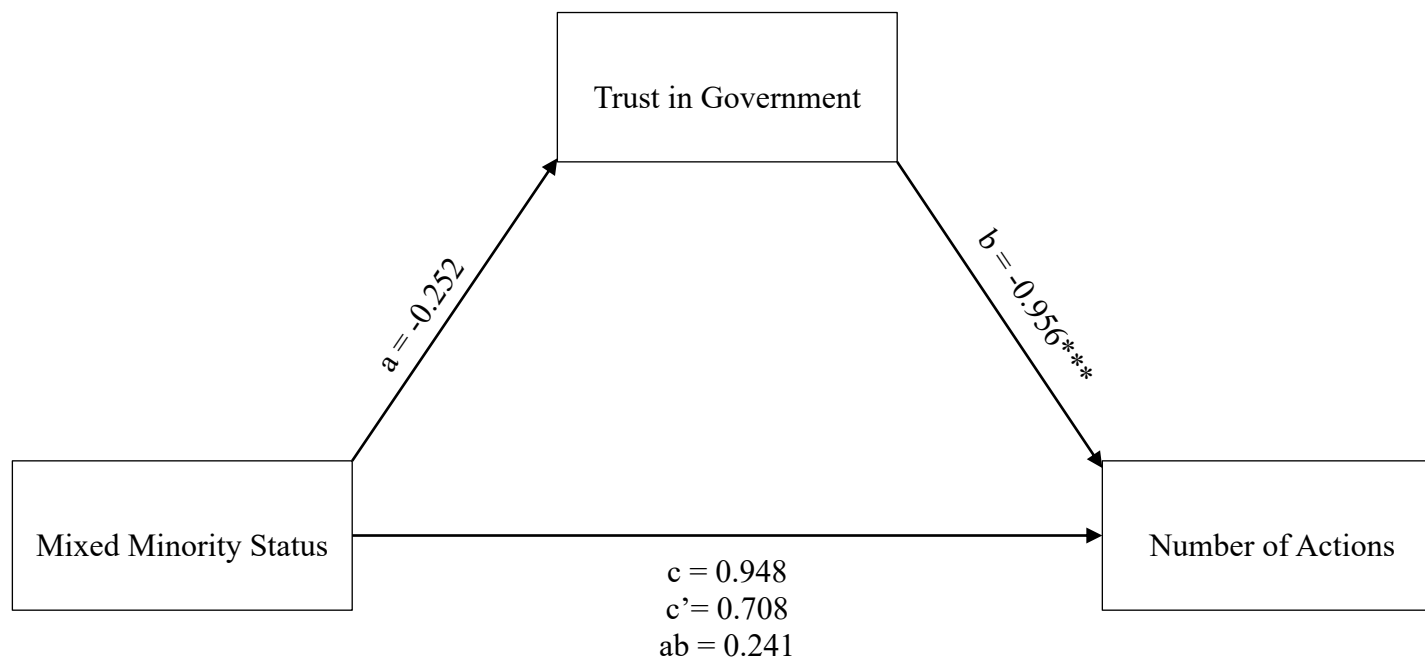

## Model 2

$N = 221$ ,  $*p < 0.05$ ,  $**p < 0.01$ ,  $***p < 0.001$ .

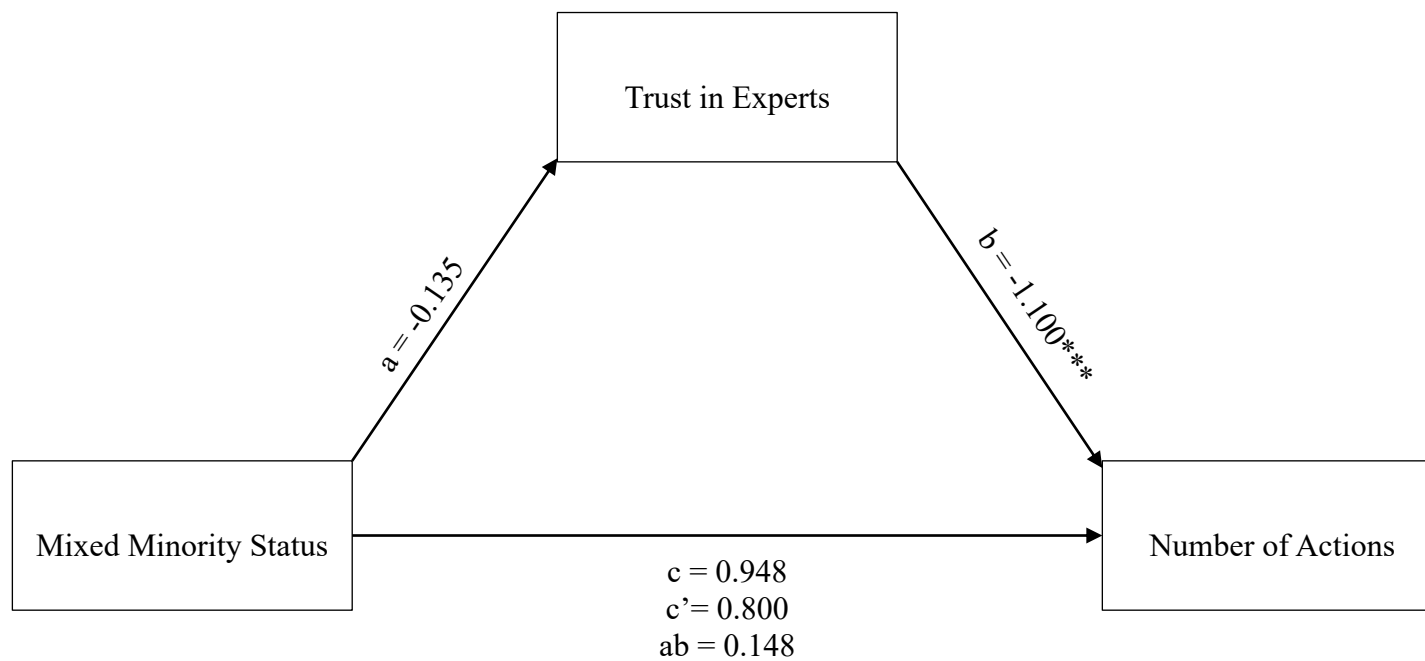

### Model 3

$N = 221$ ,  $*p < 0.05$ ,  $**p < 0.01$ ,  $***p < 0.001$ .

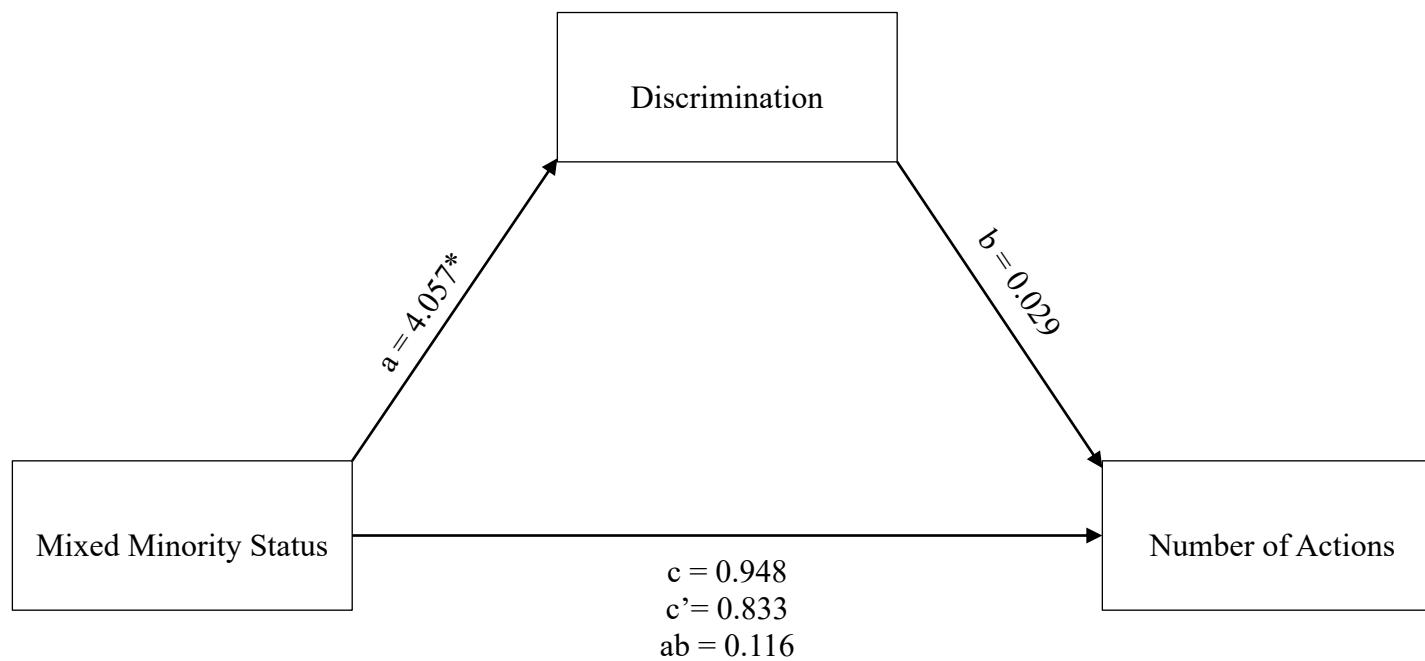

## Model 4

$N = 221$ ,  $*p < 0.05$ ,  $**p < 0.01$ ,  $***p < 0.001$ .

Supplement: S1 Fig — (PDF) [file pgph.0003871.s003.pdf]
