# Supplementary material for: Medical mistrust in racial minorities during the COVID-19 pandemic: Attitudes, actions and mental health outcomes
Source: PLOS Glob Public Health. 2024 Dec 13;4(12):e0003871. doi: 10.1371/journal.pgph.0003871 (PMC11642957; doi:10.1371/journal.pgph.0003871)
Supplement: S1 File — (PDF) [file pgph.0003871.s004.pdf]

Start of Block: Trust

Trust in Leadership How much trust do you have in White House leaders such as the President of the United States for information and guidance on COVID-19? Answer below using the scale.

- 0 - A great deal (0)
- 1 - A fair amount (1)
- 2 - Not very much (2)
- 3 - Not at all (3)

Trust in Experts How much trust do you have in a) health agencies; b) healthcare providers; scientists and researchers; c) for information and guidance on COVID-19? Answer below using the scale.

- 0 - A great deal (0)
- 1 - A fair amount (1)
- 2 - Not very much (2)
- 3 - Not at all (3)

PS How likely is it that COVID-19 will directly impact you? Answer below using the scale.

- 0 - Extremely (0)
- 1 - Very (1)
- 2 - Somewhat (2)
- 3 - A little (3)
- 4 - Not at all (4)

Perceived Severity Which one of these statements reflects your thinking about COVID-19 today? Answer below using the scale.

- 0 - I don't know what the fuss is about, this is just another flu (0)
- 1 - I have some concerns about the situation but am watching and waiting for now (1)
- 2 - I am very nervous and am trying to minimize my risk of getting sick as much as possible (2)
- 3 - I feel very panicked about this situations and cannot envision a positive ending (3)

Perceived Benefit Perceived Benefits: How important do you think it is to take specific actions to avoid COVID-19? Answer below using the scale (0 - 4: Extremely, Very, Somewhat, A little, Not at all).

- 0 - Extremely (0)
- 1 - Very (1)
- 2 - Somewhat (2)
- 3 - A little (3)
- 4 - Not at all (4)

# of Actions Taken Have you taken any of the actions below in response to the COVID-19 pandemic?

	Yes (1)	No (0)
Washing hands more often (1)	○	○
Using hand sanitizer more often (2)	○	○
Wearing a mask when going out (4)	○	○
Wearing gloves when going out (5)	○	○
Avoiding shaking hands (6)	○	○
Avoiding eating in restaurants (7)	○	○
Avoiding takeout or delivery from restaurants (8)	○	○
Avoiding travel to COVID-19 infected areas (9)	○	○
Avoiding travel by airplane (10)	○	○
Avoiding public transportation (11)	○	○
Avoiding large gatherings of people (12)	○	○
Staying home (e.g., not going to school or work) (13)	○	○

End of Block: Trust
